# Alveolar Abscess

**Published:** 1876-02

**Authors:** W. C. Barrett


### DENTAL REGISTER.

Vol. XXX.] FEBRUARY, 1876. [No. 2.

### ALVEOLAR ABSCESS.

BY W. C. BARRETT, M. D. S.

*A paper read before the Ontario Dental Society at Hamilton, Canada, July 22d, 1875.*

Alveolar abscess is the resultant of uncontrolled, disorganizing inflammation. And yet inflammation, though a morbid condition, is a step in the great reparative process of nature. The antecedent circumstances of alveolar abscess, are usually, First, inflammation, as the result of some irritation. Unchecked, it soon reaches the point of strangulation of the pulp, and this must end in death and disorganization. To eliminate the now effete matter, it is necessary that destructive metamorphosis should go on till it reaches the surface and thus permit the escape of the disintegrated tissue. But inflammation, if it attain only the proper stage, should not result in any of these ulterior consequences. Let us take as an instance, any inflamed pulp, and trace it through the succeeding changes, noting by the way such ministers and measures as might, if employed, conduce to a return to a true pathological condition.

Suppose the pulp from some cause, subjected to the action of an irritating agent. Perhaps it was a blow, possibly some morbid condition of the oral cavity, or more frequently because deprived of its protecting dentine through caries. The first result is increased vascularity, puffing up of the tissue from injection, and consequent heat, tension and swelling. The capillaries of the tissue are thus contracted, and thus free circulation is interrupted; effete matter is not eliminated, and the pulp is engorged. You may ask why is there an increased flow of blood. It may be accounted as a wise provision of nature for the healing of injuries, by carrying to the spot an augmented supply of material for the building up of the injured tissue; for we know the nutritive material is distributed throughout the system by means of the blood. Now if just sufficient action be excited to carry a proper supply of plasma, or nutritive material to the spot, the breach is quickly repaired. But the danger is that too much will be done, and right here is the first opportunity for the dentist to interfere, provided the case comes into his hands.

### INFLAMMATION OF THE PULP.

Symptoms:—Sensitiveness of the tooth to thermal changes—heat and cold—and to acids; a dull, throbbing pain, which is increased at night and upon lying down; with occasional shooting, lancinating thrills. The principal symptom to be depended upon, is the action of the cold water in mouth, which at certain stages will quiet the pain.

Treatment:—Whatever will arrest vascularity; as the continued application of cold, depletion, venesection and nauseation. Of therapeutical agents prescribe such as will reduce the violence of circulation. The best of these I have found to be, *Tincture Aconitum Napellus* or the tincture of aconite root, which acts by distending the capillaries. Apply it to the cavity (if there be one) on a pledget of cotton. Bathe the adjacent parts with dilute aconite, and avoid heating the blood for a few days.

If the inflammation be not arrested at this point, it proceeds to strangulation; that is, the blood vessels entering the pulp become so engorged and tumefied, that at the foramen they are choked by the bony walls in which they are enclosed, and so all communication with the pulp is cut off, and it dies. Now right here the moisture of the pulp may be absorbed, and it become dessicated and mummified, and so no further trouble be given. If this be not the case, the tissue is disorganized—broken down—putrefies; and in this process of disintegration it of course gives off gases and a fetid odor; and is changed into a festering mass. If a cavity be now opened into the seething pulp from outside, through the tooth, and it be kept open, the gases and festering matter may pass out without causing further pain or inconvenience; these with the dried up pulps, are the teeth which we find dead, without having been the cause of pain. But if the seething mass be securely confined within the bony walls, it seeks egress somewhere. There is no other escape than through the canal occupied by the blood vessels and nerves, which kept up the communication of the pulp with the rest of the system. The gases press through the foramen, and immediately come in contact with the membrane lining the alveolar socket in which the tooth is set. From this new source of irritation, the periosteal membrane, if not already diseased, through contact with the morbidly excited pulp, becomes inflamed, and a new set of symptoms intervene. From inflammation of the pulp, (pulpitis) where the disease was first located, we now have inflammation of the periosteum.

### PERIODONTITIS.

Symptoms:—At first an uneasiness of the tooth, which is at times extremely difficult to locate; succeeded by a soreness which is easily determined, with a dull, continuous, throbbing pain. The tooth feels as though it were elongated and touched its antagonist before the others met; which indeed it does. If the inflammation be not arrested by some kind of antiphlogistic or local treatment, phlegmonous swelling supervenes, more or less violent, which ends in a disintegration of tissue, till finally the broken down and festering mass forces its way to the surface, and gives temporary relief. A fistulous opening is formed, and the inflammation has reached the *suppurative stage*. We have now what is commonly denominated an ulcerated tooth. Let us enquire what is the precise morbid condition of the diseased part, and we may then perhaps be the better enabled to comprehend the proper treatment.

I have already said that inflammation, though a morbid condition of itself, was a part of the process of reparation. It is attended with an increased flow of blood to the diseased part, and a deposition of plasma. This plasma contains just what is needed to build up new cells.

We hear of the sac which is about the end of the tooth. In extracting a so called ulcerated tooth, you have frequently brought out a kind of diseased, tumefied mass, adherent to the tooth, bathed in pus, and which, perhaps, you held up to your patient and indicated as the cause of all the suffering; which it was not. It was the effect. This mass is laid down by some authors as *a pus secreting sac*. Pardon my seeming temerity, when I say that in my opinion a greater misnomer could not have been originated.

Pus is an exudation, the resultant of uncontrolled, destructive metamorphosis. It is never a secretion. It is dead blood and that of alveolar abscess is simply the dead liquor sanguinis, floating in which are the dead blood corpuscles, and the broken down abortive cells, that, owing to morbific action have not been vivified. It is miscarriage of a tissue cell, which comes into the world prematurely, without sufficient vitality to withstand the shock. The infant cell was aborted. The mother cell did not go its full time of gestation, and the poor progeny died. Or perhaps it was starved to death from defective nutrition. At any rate it died, and floating in a foul waste of sloughed plasma and serum, it is wept into the world a decaying corpse, offensive to every sense. This, to my apprehension, is the pathogenesis of alveolar abscess. The sac is nothing but the wall of plasma, within which, function has endeavored to circumscribe and confine the diseased locality. As you would dig a trench about a fire to isolate the flames, or as you would pour water upon it, to extinguish them, so has nature by means of the increased vascularity, wept over the morbid place a rain of plasma. This it is intended should be built up into vivific cells, and so become living tissue. But the inflammation is so great that repair can not go on. The fire must first be subdued.

Now this process of cell building, is the pivotal point upon which the whole treatment of alveolar abscess hinges, and I wish to make it plain; so permit me to amplify.

You have all of you perhaps, noted the way in which the cavity left upon extraction of a tooth—the socket in which it set—is filled up. If you have not, I would advise you to get some favorable case under your observation, and then watch it closely to its full solution. It is not every tooth extracted that will give you a good example. So if you can not in one case trace the process, take another. The first deposit in the cavity will be coagulated blood—the clot which soon forms. In twenty-four hours or less, the edges of the lacerated tissue are inflamed, though they look not red and angry, but red and moist. The clot of blood is soon sloughed out, and the cavity fills with a kind of whitish coagulum. This gradually becomes of a firmer consistency and at the end of a few days begins to assume a pinkish hue. Try it with an excavator now, and it seems to be a kind of solidified gelatine. Try it again next day, and you will find that, while it cuts like a curd, —easily, the instrument scarcely meeting resistence—blood flows from it. A day later it has assumed a firmer consistency, and blood flows from it still more freely when it is wounded. After another day or so, you will see that it has assumed the appearance of connective tissue;—of flesh; but it is the flesh of the new born infant, which, however, soon becomes like the rest of the gums.

Now what was the process which produced this series of phenomena?

First, plasma was poured out—the thin jelly like matter. This is the material of which cells are built. Proliferous cells have the power of generating within themselves a new cellular life; and thus, the plasma, in immediate contact with a healthy cell upon the border of the wound, by the creative power of vitality, arranges its particles—its proximate principles—into the form of a cell, which is vivified;—given life and physiological action by the complete cell upon the margin, and which new cell in turn is the formative parent of others, till consecutively, the whole mass is arranged into living cells. Veins, arteries, capillaries and nerves, ramify it in every direction; for it is a part of the wise provision of nature, that each cell is vivified after its kind; and thus we have connective tissue, muscular, nerve, cartilaginous and osseous tissue. This is the manner of generation.

But suppose the inflammation be too great, or the tone of the system too low for these cells when formed to be vivified or given life? In that case the cell instead of becoming tissue, is so much dead, effete matter, and is sloughed off. Its place is supplied with another, which in turn, sloughs; and these cells, floating in the pus, decaying, disintegrating, putrefying with their continual succession of abortive life, are thrown off by constant discharge.

This is *suppuration* or the suppurative stage of inflammation. If the inflammatory symptoms become more violent, and the diseases spread still further, it may attack the vital and active cells, and destroy their life; causing them in turn, to slough. This is the dangerous *ulcerative stage* of inflammation or *sphacelus.*

Now to apply these principles to our suppurative tooth. About the diseased point nature pours in plasma, and an effort is made to construct it into tissue. But there is a continual irritant there, which keeps up the exasperation and the inflammation is exacerbated. This foreign provocative is the dead and decaying pulp. Yet function is incessantly busy pouring in plasma, which but feeds the fire. Nature is a machine set to do a certain work and which never ceases to perform that labor while the possibility remains. She is an automaton, which occasionally needs the regulating hand of the physician. So she builds with the plasma, a wall of demarcation about the diseased spot, the inner portion of which consists of devitalized, abortive, broken down, sloughing attempts to build up the burned district by means of cells. The outer part is the freshly deposited plasma, and the whole mass is the ulcer, the sac, which you sometimes bring away with the extracted tooth.

Now having got to the bottom of the matter, and determined to our own satisfaction just what is the cause and what the process of both the progressive and retrograde metamorphosis, we can see our way clear to the proper solution of our problem. The method of treatment is now plain, and the first step is to remove the irritating cause. Drill into the tooth, and take out the dead pulp. Sometimes this is sufficient to effect a cure without further treatment. But if the inflammation have become chronic, other measures must be resorted to.

To go back to the first stages of periodonteal inflammation. What shall be the treatment? I mean before suppuration has been reached, and when the symptoms are the soreness and elongation of the tooth, caused by the inflammation and thickening of the investing membrane.

First remove the cause—the dead pulp. Next, use an antiphlogistic regimen, and finally reduce the temperature below the point of inflammation. The most effective application I ever tried, is ether spray, carried to the point of local insensibility. I use this in cases of violent periodontitis, when I can not well wait the action of medicine, or when the attack is so acute as to make the pain unbearable; and I seldom fail of favorable results, whether the difficulty be caused by a dead pulp, or the irritation consequent upon filling nerve canals. I use the Richardson spray apparatus, with common sulphuric ether, and deem it worth all the other local applications in the world.

If the patient do not present himself till this stage be passed, and the presence of pus be diagnosed, other treatment must be adopted. The dead, broken down cells must be removed, and healthy granulation established. How? By first cleansing the parts with tepid water, then thoroughly cauterizing the diseased surface, for the purpose of destroying—burning up the morbid tissue cells. This cauterizing may be done with the most convenient escharotic.

The actual cautery of course would be most effectual; nitrate of silver would be next best, but on account of the difficulty of access, both these agents are inadmissible, unless the last be used in solution. Creosote is a useful cautery. If there be a fistulous opening through the gums, you may, having cleaned out the pulp chamber and nerve canal, wrap a little cotton around a nerve broach, and using that as a piston, force creosote through the tooth till it appears at the fistulous opening. If this be thoroughly done, you may fearlessly fill the nerve canal immediately, to prevent further irritation. The diseased surface being removed, there will be a new deposit of plasma which will produce new, cells and you may now take care that the inflammation does not get beyond control, which if you understand its treatment, there need be no danger of.

But if there be no fistulous opening and the matter is being discharged down or up through the tooth, the method of procedure is different. If you stop the cavity so that the pus can not be discharged, you will cause such violent inflammation and pain as to be perhaps insupportable. There are two ways of going to work. One is to persist in closing the nerve canal, and thus force the pus to make its way through the alveolar walls, and form the fistulous opening, when its treatment is as previously described. The other is less painful to the patient, but more troublesome to you, and consists in persevering efforts in the applicaton of detergents and antiseptics; chloride of zinc, carbolic acid, chlorate of potassa, salicylic acid, etc. These may be inserted upon loose pledgets of cotton, while the absorbents may be stimulated by painting over the seat of disease with tincture iodine.

I have secured a resolution in some cases, by stopping up the tooth till the pain was quite severe and swelling had commenced, and then by means of the proper remedies, supplemented by the ether spray, I have secured a quick resolution of the difficulty, and healthy action. This method is strictly according to pathological rules. Time forbids my detailing the therapeutical and physiological action. I will give the process subsequently, if any desire it.

In some chronic cases of alveolar abscess of long standing, I have not waited for healthy action to supervene. In many such, the foramen of the root is closed, and remedies can not be forced through the tooth. In these instances I have filled the nerve canal and left the cure to time, which I may safely do when I have removed the irritating cause, by first filling the nerve canal securely.

At other times I have been obliged to treat from the outside through the fistulous opening. There is not the necessary time for me to detail my practice in these circumstances The proper treatment will readily occur to you, if you perfectly understand the theory of inflammation. But you will not meet with success unless you have first mastered this, and then made yourselves acquainted with the nature and effects of the remedies you use. Don't apply creosote to arrest inflammation; nor iodine, nor carbolic acid, as many have done. Creosote is an antiseptic and cauterant, and therefore. the cause of inflammation. It can only cure by metostisis. It is the same with carbolic acid and all the list of detergents, and cauterants. Tincture iodine simply excites the absorbents, and is only useful when there is work for those organs. Tincture aconite has the power to lessen inflammatory action through its potentiality in dominating neural action and thus dilating the capillaries. If then. you are not master of the theory of disease and the action of remedies, lose no time in commencing the study of pathology and therapeutics; that having a clear idea of what you wish to do, and an intimate acquaintance with your implements, success may be certain.

